# Identification of the New GmJAG1 Transcription Factor Binding Motifs Using DAP-Seq

**DOI:** 10.3390/plants13121708

**Published:** 2024-06-20

**Authors:** Jinxing Wang, Zigang Pu, Weiyao Zhang, Mengnan Qu, Lusi Gao, Wenjing Pan, Yanan Sun, Chunxu Fu, Ling Zhang, Mingkun Huang, Yufang Hu

**Affiliations:** 1 Suihua Branch of the Heilongjiang Academy of Agricultural Sciences, Suihua 152052, China; wjxsuihua@126.com (J.W.); kzwy008@163.com (W.Z.); qumengnan1988@163.com (M.Q.); gaolusi1989@163.com (L.G.); wadrjwpp@163.com (W.P.); synbmx@163.com (Y.S.); wxyfcx1976@126.com (C.F.); 2 Jiangxi Provincial Key Laboratory of Plant Germplasm Innovation and Genetic Improvement, Lushan Botanical Garden, Jiangxi Province and Chinese Academy of Sciences, Jiujiang 330022, China; pzgcl@163.com (Z.P.); linzh00@126.com (L.Z.); 3 Heilongjiang Longke Seed Industry Group Co., Ltd., Harbin 150000, China

**Keywords:** *Glycine max*, GmJAG1, DAP-seq, motifs

## Abstract

Interaction between transcription factors (TFs) and motifs is essential for gene regulation and the subsequent phenotype formation. Soybean (*Glycine max*) JAGGEED 1 (GmJAG1) is a key TF that controls leaf shape, seed number and flower size. To understand the GmJAG1 binding motifs, in this study, we performed the GmJAG1 DNA affinity purification sequencing (DAP-seq) experiment, which is a powerful tool for the de novo motif prediction method. Two new significant GmJAG1 binding motifs were predicted and the EMSA experiments further verified the ability of GmJAG1 bound to these motifs. The potential binding sites in the downstream gene promoter were identified through motif scanning and a potential regulatory network mediated by GmJAG1 was constructed. These results served as important genomic resources for further understanding the regulatory mechanism of GmJAG1.

## 1. Introduction

Recognition of specific conserved *cis*-regulatory elements (e.g., motifs) in promoters or enhancers by specific transcription factors (TFs) is essential for gene regulation, which subsequently affects the organism phenotype [1,2]. Due to the rapid development of next-generation sequencing, high-throughput sequencing data facilitated the discovery of TF motifs [3]. To date, thousands of conserved motifs have been deposited in public databases such as TRANSFAC [4], JASPAR [5], and some plant-specific motif databases like PlantTFDB [6] or PlanPAN [7], etc. Typically, each TF family recognizes a collection of similar conserved DNA sequences, whose binding presence can be represented as the motif position weight matrices (PWMs). However, the current catalog of TF binding motifs remains incomplete, making it difficult to understand the TF regulatory network, especially for a robust TF with a favored phenotype, such as the soybean (Glycine max) JAGGED 1 (GmJAG1), a C2H2 type zinc finger (C2H2-zf) TF [8,9].

GmJAG1 is located at the famous *Ln* loci controlling multiple developmental process in soybean, such as leaf shape, seed number, flower size, etc. [9,10]. The specific GmJAG1 binding motifs are far from being understood and, recently, we identified a new GmJAG1 motif (named Motif0 [M0] in this study) with core sequence GTTGGA from GmJAG1 chromatin immunoprecipitation coupled with high-throughput sequencing (ChIP-seq) data which are largely different from other TF motifs in public databases [11]. The fact that TF may have multi-target motifs has been reported [12]; however, as a key TF in soybean, it is still unknown whether GmJAG1 is able to recognize other conserved motifs. Although the GmJAG1 ChIP-seq data are available, it still has several limitations for new motifs discovered using these ChIP-seq data [13,14]. One of the major drawbacks is that the ChIP-seq was performed in vivo, which means that TF binding to the enriched peak region could occur through other interacting TF partners [13], which is supported by the observation of enrichment of other TF binding motifs such as ERF, bHLH, etc., in ChIP-seq data [11]. Using these “indirect” GmJAG1 binding regions as input for motif prediction would greatly influence the discovery of novel motifs or lead to the discovery of other TF motifs that are not bound by GmJAG1.

By contrast, DNA affinity purification sequencing (DAP-seq) is able to pull down the DNA fragments directly in vitro, avoiding the binding noise from other interacting TF partners [15]. To identify some new GmJAG1 binding motifs, we apply the DAP-seq using in vitro expressed GST-tagged GmJAG1 protein. Finally, we successfully identified two new GmJAG1 motifs (named Motif1 [M1] and Motif2 [M2]) which were further confirmed by the subsequent Electrophoretic Mobility Shift Assay (EMSA) experiments, suggesting a multiple targeted role of GmJAG1 to soybean genome and partially supporting a multiple function of GmJAG1 in the developmental process of soybean.

## 2. Results

### 2.1. Summary of GmJAG1 DAP-Seq Data

**Figure 1:**
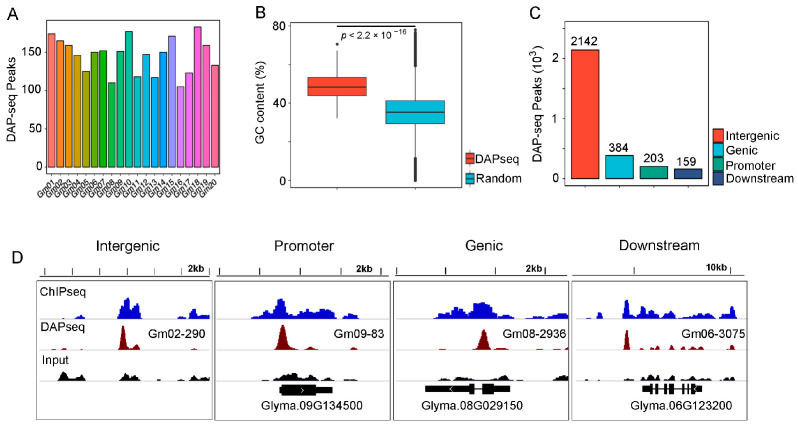
Summary of the GmJAG1 DAP-seq data. (**A**) Number of GmJAG1 enriched peaks of DAP-seq data in each chromosome. (**B**) Comparison of GC content of GmJAG1 DAP-seq enriched region and the random genomic soybean sequences. The *p* value was calculated by the Wilcoxon test. (**C**) Distribution of GmJAG1 DAP-seq enriched peaks in soybean genome. (**D**) IGV screenshot showed four GmJAG1 DAP-seq enriched peaks: Gm02-290, Gm09-83, Gm08-2936 and Gm06-3075. GmJAG1 ChIP-seq data was downloaded from a previous study.

To confirm whether GmJAG1 is able to bind some new motifs, we performed GmJAG1 DAP-seq experiments, generating DAP and input libraries. After sequencing, we obtained 73 million (M) and 40 M reads for the DAP and input libraries, respectively (Table S1). Both the GmJAG1 DAP-seq and the input control libraries show a high mapping rate (>90%) against the soybean reference genome (Table S1). In total, 2750 GmJAG1 highly enriched regions were identified (we chose an 8-fold enrichment cut-off for peak calling via HOMER v4.11 software). Approximately 100–200 peaks were distributed across each chromosome (Figure 1A, Table S2). The average GC content of these GmJAG1 enriched peaks (48.7%) is significantly higher than the random genomic sequence (34.6%) (*p*-value < 2.2 × 10^−16^) (Figure 1B), indicating that GmJAG1 prefers a high GC sequence. Furthermore, we observed that 2142, 384, 203 and 159 enriched peaks located in the distal intergenic, genic, promoter and downstream regions, respectively (Figure 1C). However, compared to GmJAG1 ChIP-seq, only 66 (2.4%) DAP-seq enriched peaks were observed that overlapped with ChIP-seq enriched peaks (Figure 1D; Table S2), indicating the large differences in GmJAG1 binding in vivo and in vitro. Nevertheless, these selectively highly enriched peak sequences by DAP-seq served as highly confident DNA input for new motif prediction using computer software (e.g., HOMER).

### 2.2. Prediction and Validation of Two New GmJAG1 Binding Motifs

**Figure 2:**
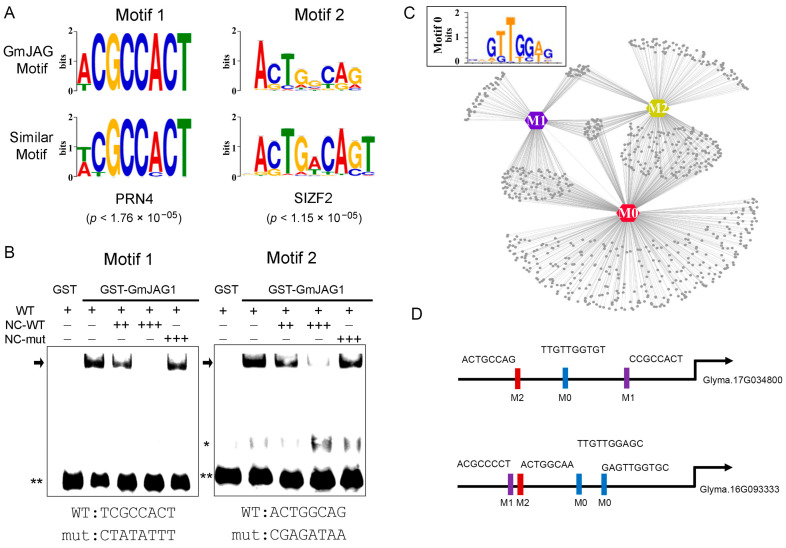
Identification of two new GmJAG1 binding motifs. (**A**) The two new predicted GmJAG1 motifs (upper panel, Motif1 and Motif2) PWM and their most similar motifs (lower panel) in the public database. (**B**) EMSA experiment confirmed the GmJAG1 binding to the Motif1 and Motif2. Non-labeled probes served as the competitor. WT, wild type probe. NC, non-labeled. Mut, mutated probe. +, which indicated 25 fmol in each lane, while ++ and +++ mean that 100× and 1000× fold of the original labeled WT probe. *, indicated non-specific binding signal and ** indicated the free probe. Arrows indicated the binding signal. The WT and mutated probe core sequences were indicated below the EMSA diagram. (**C**) Examples of potential GmJAG1 targeted genes predicted by the motif scanning. M0, M1 and M2 indicated the previously reported GmJAG1 binding motif (indicated in the black box) as well as Motif1 and Motif2 identified in this study. (**D**) Examples of match sequences of three GmJAG1 binding motifs, Motif0 (M0), Motif1 (M1) and Motif2 (M2) in the promoter of genes (e.g., Glyma.17G034800 and Glyma.16G093333).

As mentioned above, the enriched DNA sequences in DAP-seq were used for motif prediction via HOMER software. We observed that two motifs with the consensus sequences, ACGCCACT and ACTGGCAG, showed high significant enrichment (*p* value < 1 × 10^−90^) compared to the background genomic sequences. These two motifs were referred as GmJAG1 Motif 1(M1) and Motif 2 (M2), respectively, which showed less similarity to the previous GmJAG1 M0 motif (E-value > 1 × 10^−5^) (Figure 2A; Table S3). We also performed EMSA experiments using GST-GmJAG1 recombinant protein and M1, M2 biotin-labeled probes. As Figure 2B shows, GmJAG1 is able to bind to these motifs, supporting the theory that M1 and M2 are the new GmJAG1 binding motifs.

Next, we scanned these three GmJAG1 binding motifs (M0, M1 and M2) in the gene promoters to predict the potential GmJAG1 target genes. In total, 11,000, 2231 and 3399 gene promoters harbored M0, M1 and M2 were found, respectively, suggesting a potential GmJAG1-mediated regulatory network (Figure 2C,D; Table S4), which also extends our knowledge of GmJAG1 recognition diversity. Since genomic editing at cis-regulatory elements could be an efficient strategy for further crop improvement [16,17,18], the identification of new GmJAG1 binding motifs and its potential bound regions in the genome served as important resources for design of the guided RNA oligos for the CRISPR/Cas9 system.

## 3. Discussion

As a key transcription factor, understanding the specific binding motifs of GmJAG1 could help to elucidate its regulatory pattern in the soybean developmental process [3,8,9]. In this study, by coupling the DAP-seq method and the EMSA experiments we successfully discovered two new binding motifs for GmJAG1, confirming the multi-targeted binding sites of GmJAG1 in the soybean genome.

Conserved DNA motifs are essential for TF recognition and identification of the specific TF motifs is the key step in interpreting the complicated regulatory network [1,2,19]. However, because the conserved motifs are often short and typically degenerate, the discovery of motifs in the plant genome remains a challenging task. In the model plant Arabidopsis, approximately 1700 TF motif collections have been deposited in the public motifs databases such as PlantPAN and PlantTFDB [6,7], representing about 50–60% of the TFs in Arabidopsis. In contrast, only about 370 out of ~4000 soybean TFs have corresponding motifs in these databases and most of them are predicted from the known motifs of the homologous TFs in other species [6]. In contrast to human or model plants (e.g., Arabidopsis), there are many databases with abundant sequencing data for understanding the function of non-coding regions, such as the human ENCODE (https://www.encodeproject.org accessed on 1 May 2024) and the Arabidopsis Cistrome database [20]. Although soybean also has abundant DNA resequencing data characterizing the single nucleotide polymorph (SNP) or structural variation (SV) at the genomic level, for example, the recently published soybean PAN-genome data [21], few high-throughput sequencing data (e.g., ATAC-seq) [22,23,24] have been aimed at finding these functional non-coding elements (e.g., motifs) in the soybean genome. Compared to the ChIP-seq method [14], a traditional strategy for discovering new motifs, DAP-seq [15] is able to select the DNA fragments directly bound by the recombinant TF in vitro, which showed some advantage over the ChIP-seq in finding motifs. Therefore, DAP-seq may be prioritized in further studies to elucidate soybean TF binding motifs, but it is a long-term undertaking. Since the variation in the non-coding region is associated with soybean domestication [16] and manipulation of these binding motifs via genome editing [17,18] is one of the efficient strategies used to improve agronomic traits, the understanding of GmJAG1 binding motifs would be integrated into large scale DNA re-sequencing data of soybean to uncover some potential functional GmJAG1 motif variation in soybean domestication.

## 4. Materials and Methods

### 4.1. Preparation of GmJAG1 DAP-Seq Library

DAP-seq library preparation was conducted according to the method described in a previous report [15]. Briefly, the full-length coding sequence of *GmJAG1* (*Glyma.20G116200*) was cloned into the *pGEX-4T-1* vector and transformed into the *E. coil* (Rosetta DE3). The GST-GmJAG1 recombinant protein was induced by 0.2 mM IPTG at 16 °C overnight and purified by the MagneGST beads (Promega, Beijing, China, cat.no.V8600). The DNA library was prepared using genomic DNA from a whole soybean plant via VAHTS Universal Pro DNA Library Prep Kit for Illumina Kit (Vazyme, Nanjing, China, cat.no.ND608) according to the manufacturer’s instructions. About 100 ng of DNA library was incubated with the beads and GmJAG1 recombinant protein mixture at room temperature for 1 h. The MagneGST beads were washed three times with 1 × PBS + NP40 (0.005%), followed by two washes with 1X PBS. After washing, the enriched DNA fragments pulled down by GST-GmJAG1 were recovered and amplified for 12 cycles using primers from the VAHTS Multiplex Oligos Set 4/5 for Illumina Kit (Vazyme, Nanjing, China, cat.no. N321). The PCR procedure was set to 98 °C for 1 min, 98 °C for 15 s, 60 °C for 20 s and 72 °C for 20 s. The final DAP-seq library was sent for Illumina sequencing in 150 bp paired-end mode. The primers are listed in Table S5.

### 4.2. DAP-Seq Data Processing and Motif Analysis

The adaptors and reads with low quality in the raw data of DAP-seq were removed by Trim_galore v6.10 software (https://www.bioinformatics.babraham.ac.uk/projects/trim_galore/ (accessed on 1 May 2024)). Then, the filtered DAP-seq reads with high sequencing quality were mapped to the Williams 82 V4 reference genome (https://phytozome-next.jgi.doe.gov (accessed on 1 May 2024)) using bowtie2 v2.54 software [25]. Mapped reads with MAPQ value over 30 were extracted by Samtools [26] and used for peak calling via HOMER v4.11 (http://homer.ucsd.edu/homer/ (accessed on 1 May 2024)) with the following parameters: findPeaks-style factor-F 8. The script “getFocalPeaks.pl” in HOMER was used to extract the enriched regions with a focal score over 0.9, which were retained for subsequent motif prediction. Motif prediction and scanning were performed using the scripts, findMotifs.pl and findMotifsGenome.pl in the HOMER software with the parameters set as default.

### 4.3. EMSA Experiment

The EMSA experiment was conducted according to a previous study [11]. Briefly, the *pGEX-4T-1* and *pGEX-4T-1-GmJAG1* vectors were transformed into the Rosetta (DE3) *E. coli* competence cell as mentioned above. The GST and GST-GmJAG1 recombinant proteins were induced with 0.2 mM IPTG at 16 °C overnight and purified by the MagneGST beads (Promega, Beijing, China, cat.no.V8600) according to the manufacturer’s instructions. The 5′-end biotinylated oligonucleotide containing the GmJAG1 putative binding motifs served as the probes. The unlabeled wild type or mutant oligonucleotides served as the cold competitors. The recombinant proteins (1 μg) were mixed with the labeled probes with or without cold competitors in 20 μL binding buffer at room temperature for 0.5 h. The protein–probes mixture was then loaded into the native gel. The gel shift assay was performed via LightShift Chemiluminescent EMSA Kit (Thermo, Waltham, MA, USA, cat.no.20148) and detected by ChemiDoc Imaging Systems (Bio-Rad, Shanghai, China). Probe sequences are listed in Table S5.

## 5. Conclusions

As a key TF involved in multiple developmental process of soybean, the binding motifs of GmJAG1 and its potential binding regions in the soybean genome are still elusive. In this study, two new GmJAG1 binding motifs were predicted by DAP-seq and their binding ability was further validated by EMSA experiments. Taken together, these findings served as important genomic resources for further understanding the regulatory mechanism of GmJAG1.

## Data Availability

All the raw data in this study can be found in The National Center for Biotechnology Information (NCBI) under the accessions, SRX11670466 (GmJAG1 DAP-seq) and SRX11670460 (DAP-seq Input).

## Supplementary Materials

The following supporting information can be downloaded at: https://www.mdpi.com/article/10.3390/plants13121708/s1, Table S1. Summary of GmJAG1 DAP-seq raw data, Table S2. GmJAG1 DAP-seq enriched peaks, Table S3. GmJAG1 binding motif information, Table S4. Motifs position at gene promoter, Table S5. Oligos were used in this study.
